# Antennal Transcriptome Analysis and Comparison of Chemosensory Gene Families in Two Closely Related Noctuidae Moths, *Helicoverpa armigera* and *H. assulta*

**DOI:** 10.1371/journal.pone.0117054

**Published:** 2015-02-06

**Authors:** Jin Zhang, Bing Wang, Shuanglin Dong, Depan Cao, Junfeng Dong, William B. Walker, Yang Liu, Guirong Wang

**Affiliations:** 1 State Key Laboratory for Biology of Plant Diseases and Insect Pests, Institute of Plant Protection, Chinese Academy of Agricultural Sciences, Beijing, 100193, China; 2 Education Ministry Key Laboratory of Integrated Management of Crop Disease and Pests, College of Plant Protection, Nanjing Agricultural University, Nanjing, 210095, China; 3 College of Forestry, Henan University of Science and Technology, Luoyang, 471003, China; 4 Swedish University of Agricultural Sciences, Department of Plant Protection Biology, Chemical Ecology Research Group, Alnarp, Sweden; University of California Davis, UNITED STATES

## Abstract

To better understand the olfactory mechanisms in the two lepidopteran pest model species, the *Helicoverpa armigera* and *H. assulta*, we conducted transcriptome analysis of the adult antennae using Illumina sequencing technology and compared the chemosensory genes between these two related species. Combined with the chemosensory genes we had identified previously in *H. armigera* by 454 sequencing, we identified 133 putative chemosensory unigenes in *H. armigera* including 60 odorant receptors (ORs), 19 ionotropic receptors (IRs), 34 odorant binding proteins (OBPs), 18 chemosensory proteins (CSPs), and 2 sensory neuron membrane proteins (SNMPs). Consistent with these results, 131 putative chemosensory genes including 64 ORs, 19 IRs, 29 OBPs, 17 CSPs, and 2 SNMPs were identified through male and female antennal transcriptome analysis in *H. assulta*. Reverse Transcription-PCR (RT-PCR) was conducted in *H. assulta* to examine the accuracy of the assembly and annotation of the transcriptome and the expression profile of these unigenes in different tissues. Most of the ORs, IRs and OBPs were enriched in adult antennae, while almost all the CSPs were expressed in antennae as well as legs. We compared the differences of the chemosensory genes between these two species in detail. Our work will surely provide valuable information for further functional studies of pheromones and host volatile recognition genes in these two related species.

## Introduction

Olfaction plays a key role in many aspects of insect behavior, such as foraging, oviposition and mate recognition. Possessing a sophisticated olfactory system to detect and interpret odorants in the environment is a prerequisite to survival and reproduction for insects. An understanding of how chemicals are detected by the antenna, transduced to the brain, and consequently translated into behavior is essential to clarify the mechanism of odorant detection in insects.

In the last few decades, much progress has been made in deciphering the mechanisms of periphery detection of insect olfaction. Olfactory signal transduction is best summarized in several discrete steps: first, the hydrophobic chemical volatiles enter into the sensillum lymph through the pores on the surface of the sensilla [1,2], and then bind to water-soluble odorant binding proteins (OBPs)/ chemosensory protein (CSPs), which are aboundunt in the sensillum lymph (up to 10 mM) [3–10]. Then, the odorants activate the odorant/ionotropic receptors (ORs/IRs) expressed on the dendritic membrane of olfactory sensory neurons (OSNs) alone or in complex with the binding proteins [11,12], upon which, the chemical signal is translated into electrical signals that are transduced to the antennal lobe (AL). In addition, sensory neuron membrane proteins (SNMPs), and odorant degrading enzymes (ODEs) are also involved in different steps in signal transduction pathway [13–16].

Identification of the chemosensory genes is prerequisite for functional exploration of olfactory genes. Previously, these studies have mostly been focused on model species with sequenced genome available, such as *Drosophila melanogaster, Bombyx mori, Aphis gossypii* and several other insect species [17–22]. With the development of the next generation sequencing (NGS) techniques, numerous chemosensory genes have been identified from various insect species, such as *Manduca sexta* [23], *Helicoverpa armigera* [24], *Cydia pomonella* [25], *Spodoptera littoralis* [26–28], *Sesamia inferens* [29], *Chilo suppressalis* [30] and *Aphis gossypii* [31], etc.

The two closely related species, *H. armigera* and *H. assulta* are important pest species in China. These two species are so similar that they both use (Z)-11-hexadecenal (Z11-16:Ald) and (Z)-9-hexadecenal (Z9-16:Ald) as their main sex pheromone components, but in nearly reversed ratios [32]. However, their foraging ranges are widely differentiated, *H. armigera* is a polyphagous species posing a major threat to over 200 different plants, while *H. assulta* is an oligophagous insect, which mainly feed on Solanaceae plants, including tobacco and hot pepper [33,34]. Their feeding preferences may be associated with differences in their olfactory and gustatory system. Aiming to understand the olfactory mechanism of these two related species, our lab previously conducted a 454 sequencing of adult male and female antennae from *H. armigera* [24]; several chemosensory genes, including 45 ORs and 12 IRs were identified. However, considering the hypothesis that the number of the glomeruli equals the number of ORs [35,36], the number of ORs was lower than expected, suggesting that some ORs might have been missed. The number of other identified chemosensory genes (GRs, OBPs, and CSPs) was also less than the number of genes reported in other Lepidoptera insects [26,37]. This might be because the sequencing depth of 454 is much lower than Illumina technology and some low-level expressed genes were omitted. In order to find the missing chemosensory genes in *H. armigera* and identify all of the olfactory genes in *H. assulta*, we sequenced the adult antennae of *H. armigera* and *H. assulta* using Illumina HiSeq 2000 platform. Our study greatly enriches the information on the molecular mechanisms of chemoreception in these species, with a total number of 133 putative chemosensory unigenes in *H. armigera* including 60 ORs, 19 IRs, 34 OBPs, 18 CSPs, and 2 SNMPs, and 131 putative chemosensory unigenes with 64 ORs, 19 IRs, 29 OBPs, 17 CSPs, and 2 SNMPs in *H. assulta*. Then we completely compared the differences of the chemosensory genes (ORs, IRs, OBPs, CSPs) between these two species. Further Reverse Transcription-PCR (RT-PCR) assays in *H. assulta* were conducted to examine the expression profile of these unigenes in different tissues. Our work will surely provide an extensive molecular basis for further research in pheromone and host volatile recognition of these two related species.

## Results and Discussion

### Transcriptome assembly and Gene Ontology (GO) Annotation

The RNA extracted from the mix of male and female antennae of *H. armigera* was sequenced using Illumina HiSeq 2000 platform, while the RNA from male and female antennae of *H. assulta* was sequenced separately. A total of 47,407,880 and 51,051,262 raw reads were obtained from male and female *H. assulta* antennae samples, respectively and a total of 58,035,052 raw reads were got from the mixed sample of *H. armigera*. After trimming adaptor sequences, contaminating sequences and low quality sequences, high quality contigs were generated. These contigs were further assembled by paired-end joining and gap-filling, and clustered into unigenes. An overview of the sequencing and assembly process is presented in Table 1. The clean reads of the three antennal transcriptomes in this study have been in the NCBI SRA database, under the accession number of SRX707455 (*H. assulta* male), SRX707456 (*H. assulta* female) and SRX707450 (*H. armigera* mix-sex). The results showed 50.8% (*H. armigera*) and 54.0% (*H. assulta*) of the unigenes were matched to the entries in NCBI non-redundant (nr) protein database by blastx homology search with a cut-off E-value of 10^−5^ (S1 Material).

**Table 1:** Assembly summary of *H. assulta and H. armigera* antennal transcriptome.

	**Sample**	**Total Number**	**Total Length(nt)**	**Mean Length(nt)**	**N50**	**Consensus Sequences**	**Distinct Clusters**	**Distinct Singletons**
***H. assulta***								
**Contig**	Female	79,148	37,642,130	476	1283	-	-	-
	Male	82,205	37,372,283	455	1227	-	-	-
**Unigene**	Female	50,763	53,062,714	1045	2270	50,763	13,100	37,663
	Male	50,698	51,544,572	1017	2205	50,698	14,234	36,464
**Merge**	All	44,319	59,006,938	1331	2488	44,319	15,830	28,489
***H. armigera***								
**Contig**	Mix	97,631	38,170,555	391	811	-	-	-
**Unigene**	Mix	53,479	48,077,592	899	1597	53,479	17,149	36,330

**Figure 1:**
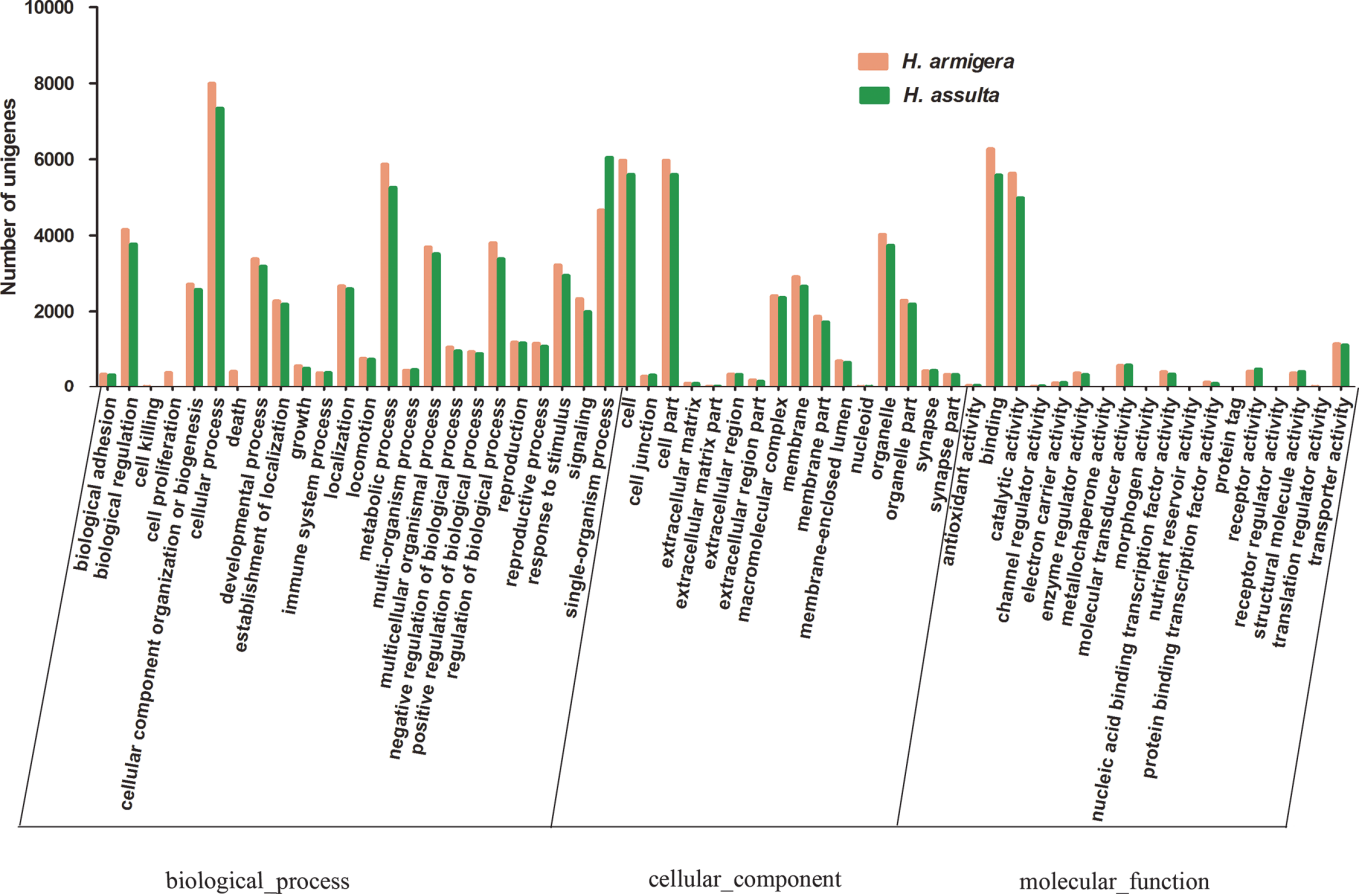
Gene ontology (GO) classification of the *H. armigera* and *H. assulta* unigenes with Blast2GO program.

Fig. 1 illustrates the distribution of the *H. armigera* and *H. assulta* unigene set in GO terms. Among the 53,479 (*H. armigera*) and 44,319 (*H. assulta*) unigenes, only 12,611 (23.6%) and 11,369 (25.6%) correspond to at least one GO-term, respectively. Similar results were observed in other transcriptome analyses for *M. sexta* [23], and *Sesamia inferens* [29]. As one unigene could align to multiple GO categories, 51,360 and 54,273 were assigned to biological process, 26,169 and 27,809 to cellular component and 14,086 and 15,489 to molecular function in *H. assulta* and *H. armigera*, respectively. In the molecular function category, the terms of binding and catalytic activity were the most represented. In the cellular component terms, cell and cell part were the most abundant. Cellular process, single-organism process and metabolic process were most abundant in the biological process category. In each of the three GO categories, the more abundant terms were almost the same as those observed in the antennal transcriptome of *M. sexta* [23], *S. littoralis* [26–28], *S. inferens* [29], *A. gossypii* [31] and *Agrotis ipsilon* [38].

### The candidate olfactory receptors in *H. armigera* and *H. assulta*

**Figure 2:**
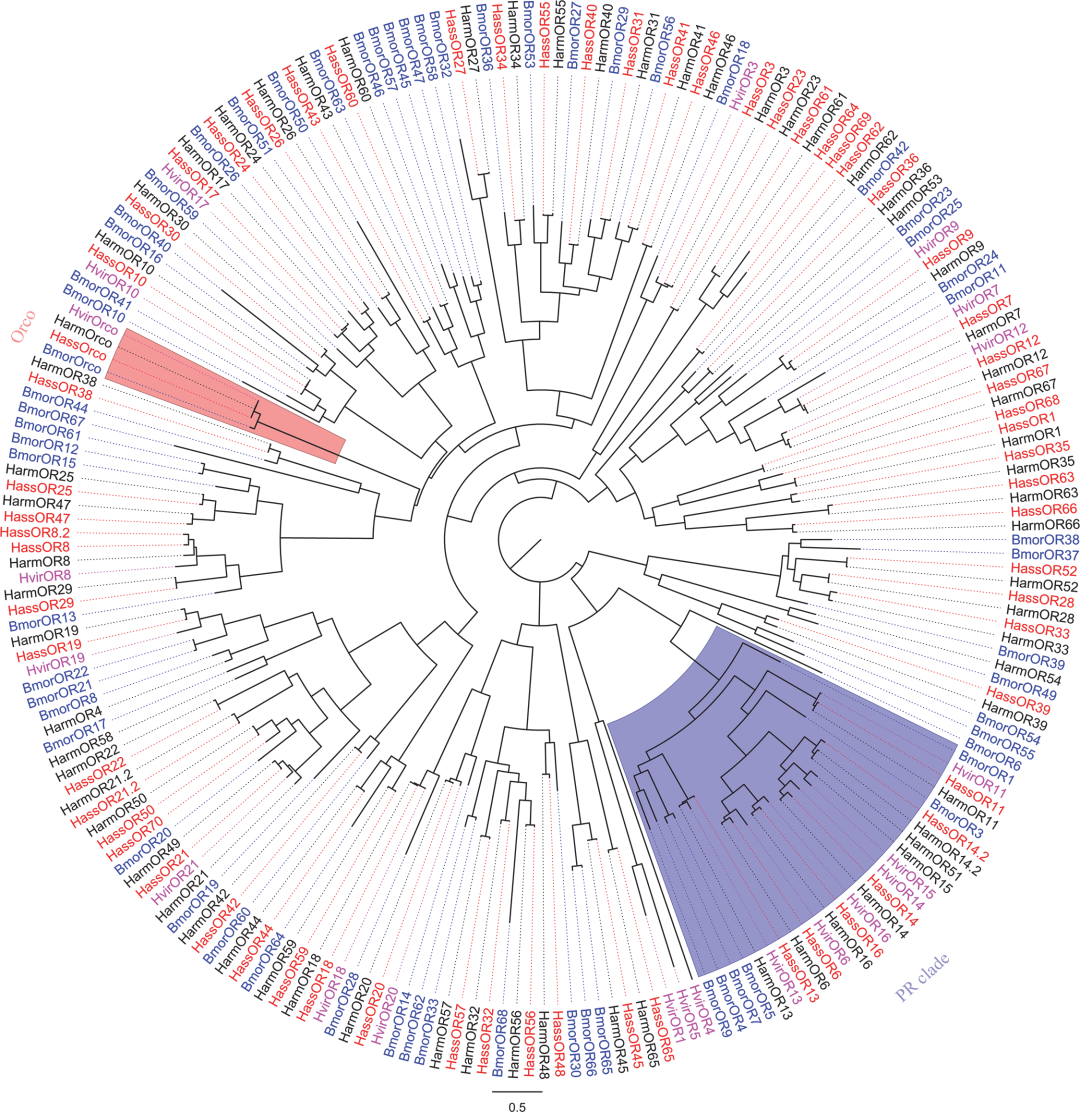
Phylogenetic tree of putative *H. armigera* and *H. assulta* ORs with other Lepidoptera ORs. This tree was constructed using FastTree based on alignment results of MAFFT. Harm: *H. armigera* (black), Hass: *H. assulta* (red), Bmor: *B. mori* (blue), Hvir: *H. virescens* (purple).

**Figure 3:**
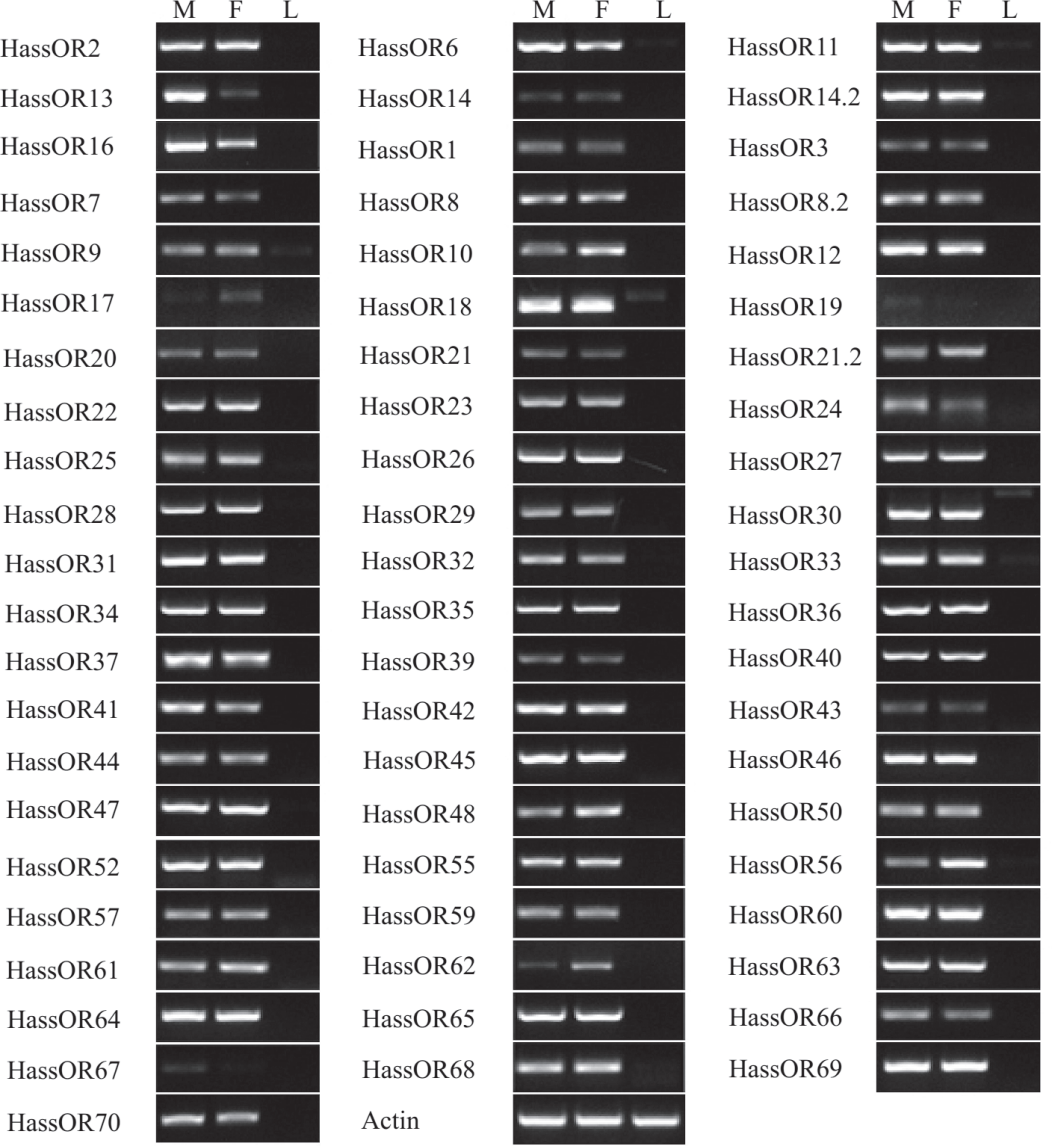
Tissue- and sex- specific expression of *H. assulta* candidate OR genes. M: male antennae, F: female antennae, L: legs (both sexes mixed).

As the centerpiece of peripheral olfactory reception, ORs are the most important and determine the sensitivity and specificity of odorant reception [16]. All of the unigenes were searched by blastx and tblastn using the ORs identified from *H. armigera* [24,39] and *H. virescens* [40], leading to identification of 60 putative OR genes in *H. armigera* and 64 OR genes in *H. assulta*, which was almost consistent with the number of glumeruli (65) in *H. armigera* and *H. assulta* [41]. All these OR genes in *H. assulta* and the ORs not identified in previous 454 sequencing of *H. armigera* were listed in Table 2. Considering the hypothesis that the number of glomeruli is equal to the number of ORs [35,36], we conclude that most of the ORs have now been identified. In the *H. armigera* antennal transcriptome, all of the 45 previously identified ORs were accounted for. HarmOR37 and OR38, which we predicted to be two genes, turned out to be two fragments of one transcript. We have identified 15 ORs from our Illumina sequencing that were not identified in the 454 sequencing and 6 of these genes have not been identified in any other previous study on *H. armigera*. One of the PR HarmOR14.2 identified by Jiang et al [42], was also found in this Illumina sequencing. The previously identified HarmOR5 was actually a GR, consistent with a report from Liu et al [39]. In *H. assulta*, 64 candidate ORs were identified. 59 of them had high similarity (>70%) with ORs from *H. armigera*, suggesting they may be orthologous genes in these two species. Notably, there were two OR8 homologies named as HassOR8 and OR8.2. In total, 5 species-specific ORs were found in *H. assulta* and named as HassOR8.2, 64, 68, 69, 70. Conversely, homologues of HarmOR15 were not found in *H. assulta* and it might be a species-specific OR in *H. armigera*. The absence of HassOR15 could suggest that this gene has low transcription in the antennae, which precluding its identification in the antennal transcriptome, or else HassOR15 may not exist in *H. assulta*. We tested for HassOR15 with PCR, but not find any bands. Transcriptome analysis of *S. littoralis* did not result in identification of a HarmOR15 homologue either [26–28]. Liu et al (2014) reported that they identified 57 ORs from *H. armigera* transcriptome, and they did not identify HarmOR28, 33, 37 in their transcriptome [39], however in our study, these genes were found, and the homologues of HassOR28, 33, 37 were also identified. Six of the HarmORs (OR4, 49, 51, 53, 54, 58) identified by Liu et al (2014) were not found in this *H. armigera* transcriptome neither in *H. assulta*. These 64 *H. assulta* ORs and 66 *H. armigera* ORs (including HarmOR4, 49, 51, 53, 54, 58) clustering with 62 *B. mori* ORs [43] and 21 *H. virescens* ORs [40,44] were used for a phylogenetic analysis (Fig. 2). In this analysis, the olfactory co-receptor (Orco) and pheromone receptor (PR) families were highly conserved, while other HassORs clustered seperately with HarmORs. Consistent with the results of sequence similarity analysis, the phylogenetic analysis also showed that there are 59 groups of orthologous OR genes in these two species. There are 7 *H. armigera* unique ORs (HarmOR4, 15, 49, 51, 53, 54, 58) and 5 *H. assulta* unique ORs (HassOR8.2, 64, 68, 69, 70). The complete comparison of the OR genes in *H. armigera* (from our study and Australia) and *H. assulta* were listed in S3 Material. Even for the PRs, which display high similarities in their sequences, their functions were somewhat different [42,45]. These differences may account for the recognition of species-specific pheromone blends. Other ORs, which had relatively low similarities compared to PRs, may be associated with detection of host plant compounds. Functional characterization of the ORs from these two species will ultimately contribute to an explanation of principles in host selection. The RT-PCR results showed that 64 ORs were exclusively or primarily expressed in the antennae, which verified the integrity of the transcriptome assembly (Fig. 3). The PR13 was biased in male antenna while other ORs were almost equally expressed in both male and female antenna, which is consistent with results reported recently [42]. Faint expressions of *OR9* and *OR18* in the legs is consistent with the existence of chemosensory sensilla on the surface of legs, as earlier study by Sun et al also reported that OR5 was expressed in male moth legs of *Plutella xyllostella* [46].

**Table 2:** Unigenes of candidate olfactory receptors in *H. assulta* and *H. armigera*.

**Unigene reference**	**Gene Name**	**Length (bp)**	**ORF (aa)**	**Blastx best hit (Reference/Name/Species)**	**E value**	**Identity**	**Transmembrane domain (No)**	**Full length**
***H. assulta***								
**Co-receptor**								
CL1541.Contig3	HassOrco	3223	473	gi|163845598|gb|ABU45983.2| odorant receptor Or83b [Helicoverpa assulta]	0	100%	7	Yes
**Pheromone receptors**								
CL613.Contig1	HassOR6	1518	425	gi|240148399|gb|ACS45306.1| olfactory receptor 6 [Helicoverpa assulta]	0	100%	7	Yes
CL373.Contig1	HassOR11	1802	431	gi|240148403|gb|ACS45308.1| candidate odorant receptor 2 [Helicoverpa assulta]	0	100%	6	Yes
CL174.Contig16	HassOR13	1596	424	gi|240148401|gb|ACS45307.1|candidate odorant receptor 1 [Helicoverpa assulta]	0	100%	7	Yes
CL4389.Contig1	HassOR14	845	244	gi|582120691|gb|AHI44516.1| olfactory receptor 14 [Helicoverpa assulta]	0	100%	4	No
CL2595.Contig3	HassOR14.2	1498	440	gi|486139852|gb|AGK90019.1| olfactory receptor 14b [Helicoverpa assulta]	0	99%	4	Yes
CL613.Contig3	HassOR16	2008	322	gi|240148405|gb|ACS45309.1| candidate odorant receptor 3 [Helicoverpa assulta]	0	100%	4	No
**Olfactory receptors**								
Unigene8904	HassOR1	1621	454	gi|148533561|gb|ABQ84982.1| putative chemosensory receptor 12 [Spodoptera littoralis]	0	84%	4	Yes
Unigene8630	HassOR3	1296	256	gi|486139730|gb|AGK90013.1| olfactory receptor 3 [Helicoverpa assulta]	0	98%	4	No
CL5377.Contig2	HassOR7	1920	411	gi|486139804|gb|AGK90015.1| olfactory receptor 7 [Helicoverpa assulta]	0	99%	5	No
CL4032.Contig2	HassOR8	1419	396	gi|22293497|emb|CAD31949.1| putative chemosensory receptor 8 [Heliothis virescens]	0	73%	6	No
CL4032.Contig3	HassOR8.2	1198	346	gi|22293497|emb|CAD31949.1| putative chemosensory receptor 8 [Heliothis virescens]	0	68%	5	No
Unigene13949	HassOR9	1339	401	gi|486139812|gb|AGK90016.1| olfactory receptor 9 [Helicoverpa assulta]	0	42%	6	Yes
Unigene3735	HassOR10	2073	390	gi|486139829|gb|AGK90017.1| olfactory receptor 10 [Helicoverpa assulta]	0	99%	4	Yes
Unigene20510	HassOR12	1462	408	gi|486139840|gb|AGK90018.1| olfactory receptor 12 [Helicoverpa assulta]	0	98%	6	Yes
CL2098.Contig1	HassOR17	1322	399	gi|486139869|gb|AGK90020.1| olfactory receptor 17 [Helicoverpa assulta]	0	99%	6	Yes
Unigene11863	HassOR18	1307	398	gi|486139883|gb|AGK90021.1| olfactory receptor 18 [Helicoverpa assulta]	0	85%	4	Yes
Unigene17037	HassOR19	1451	402	gi|51127350|emb|CAG38120.1| putative chemosensory receptor 19 [Heliothis virescens]	3.00E-177	51%	6	Yes
CL771.Contig3	HassOR20	1552	393	gi|486139895|gb|AGK90022.1| olfactory receptor 20 [Helicoverpa assulta]	0	99%	7	Yes
Unigene1626	HassOR21	1535	403	gi|51127354|emb|CAG38122.1| putative chemosensory receptor 21 [Heliothis virescens]	0	84%	7	Yes
CL117.Contig1	HassOR21.2	1293	408	gi|452113244|gb|AGG08879.1| putative olfactory receptor 19 [Spodoptera litura]	9.00E-156	58%	6	No
Unigene20296	HassOR22	1367	414	gi|452113244|gb|AGG08879.1| putative olfactory receptor 19 [Spodoptera litura]	3.00E-79	38%	5	Yes
Unigene13967	HassOR23	1262	413	gi|333408659|gb|AEF32141.1| odorant receptor [Spodoptera exigua]	0	81%	7	Yes
Unigene1383	HassOR24	1303	391	gi|114217265|dbj|BAF31195.1| candidate olfactory receptor [Bombyx mori]	1.00E-174	68%	4	Yes
Unigene7768	HassOR25	1322	390	gi|152963569|tpg|DAA05974.1| TPA: odorant receptor 15 [Bombyx mori]	3.00E-92	46%	7	Yes
Unigene12785	HassOR26	2241	408	gi|452113238|gb|AGG08876.1| putative olfactory receptor 51 [Spodoptera litura]	0	57%	4	Yes
Unigene10865	HassOR27	1578	427	gi|550848946|gb|AGY14581.1| putative odorant receptor, partial [Sesamia inferens]	5.00E-120	48%	6	Yes
Unigene9858	HassOR28	1315	392	gi|390276101|gb|AFL70813.1| odorant receptor 50, partial [Manduca sexta]	8.00E-146	59%	5	No
Unigene18117	HassOR29	1307	395	gi|238623677|dbj|BAH66312.1| olfactory receptor [Bombyx mori]	7.00E-128	44%	7	Yes
Unigene21012	HassOR30	2349	382	gi|238623681|dbj|BAH66314.1| olfactory receptor [Bombyx mori]	0	48%	6	Yes
Unigene20285	HassOR31	1313	395	gi|550848964|gb|AGY14590.1| putative odorant receptor, partial [Sesamia inferens]	0	69%	6	Yes
Unigene18165	HassOR32	1345	393	gi|152963599|tpg|DAA05989.1| TPA_exp: odorant receptor 33 [Bombyx mori]	2.00E-113	42%	4	No
Unigene2633	HassOR33	3736	414	gi|390276101|gb|AFL70813.1| odorant receptor 50, partial [Manduca sexta]	4.00E-139	52%	6	Yes
Unigene5593	HassOR34	1318	393	gi|238623718|dbj|BAH66333.1| olfactory receptor [Bombyx mori]	4.00E-169	60%	6	Yes
CL5009.Contig1	HassOR35	1617	407	gi|238623728|dbj|BAH66338.1| olfactory receptor [Bombyx mori]	1.00E-24	27%	5	Yes
Unigene7932	HassOR36	2330	387	gi|238623728|dbj|BAH66338.1| olfactory receptor [Bombyx mori]	1.00E-154	65%	7	Yes
Unigene463	HassOR38	1390	429	gi|452113240|gb|AGG08877.1| putative olfactory receptor 44 [Spodoptera litura]	0	89%	5	Yes
CL1016.Contig2	HassOR39	1244	387	gi|550848962|gb|AGY14589.1| putative odorant receptor, partial [Sesamia inferens]	6.00E-114	66%	6	No
CL2958.Contig1	HassOR40	1468	401	gi|238623701|dbj|BAH66324.1| olfactory receptor [Bombyx mori]	9.00E-132	66%	7	No
CL3469.Contig2	HassOR41	2067	402	gi|238623753|dbj|BAH66350.1| olfactory receptor [Bombyx mori]	0	75%	6	Yes
CL3125.Contig2	HassOR42	2949	445	gi|238623761|dbj|BAH66354.1| olfactory receptor [Bombyx mori]	0	69%	6	Yes
CL3068.Contig1	HassOR43	1726	396	gi|238623768|dbj|BAH66358.1| olfactory receptor [Bombyx mori]	3.00E-133	51%	7	Yes
Unigene5665	HassOR44	1344	415	gi|550848960|gb|AGY14588.1| putative odorant receptor, partial [Sesamia inferens]	5.00E-98	54%	7	Yes
Unigene20272	HassOR45	1481	429	gi|379070042|gb|AFC91732.1| putative odorant receptor OR24 [Cydia pomonella]	5.00E-166	58%	7	Yes
Unigene14231	HassOR46	1477	448	gi|550848938|gb|AGY14577.1| putative odorant receptor, partial [Sesamia inferens]	0	69%	6	Yes
Unigene6202	HassOR47	1287	179	gi|152963569|tpg|DAA05974.1| TPA: odorant receptor 15 [Bombyx mori]	9.00E-64	45%	2	No
Unigene1397	HassOR48	1183	322	gi|152963593|tpg|DAA05986.1| TPA_exp: odorant receptor 30 [Bombyx mori]	6.00E-117	56%	3	No
CL2112.Contig2	HassOR50	1386	383	gi|666916203|gb|AIG51896.1| odorant receptor, partial [Helicoverpa armigera]	0	98%	6	No
Unigene14012	HassOR52	1466	419	gi|666916207|gb|AIG51898.1| odorant receptor [Helicoverpa armigera]	0	99%	7	Yes
Unigene14277	HassOR55	1709	405	gi|666916213|gb|AIG51901.1| odorant receptor, partial [Helicoverpa armigera]	5e-151	98%	3	Yes
Unigene21491	HassOR56	1311	395	gi|666916215|gb|AIG51902.1| odorant receptor, partial [Helicoverpa armigera]	0	97%	7	No
Unigene14960	HassOR57	1283	390	gi|666916217|gb|AIG51903.1| odorant receptor, partial [Helicoverpa armigera]	0	98%	7	Yes
CL4114.Contig1	HassOR59	1670	416	gi|666916221|gb|AIG51905.1| odorant receptor, partial [Helicoverpa armigera]	4e-169	98%	6	Yes
CL1839.Contig1	HassOR60	1463	391	gi|666916223|gb|AIG51906.1| odorant receptor [Helicoverpa armigera]	0	98%	5	No
Unigene8759	HassOR61	1490	343	gi|270012723|gb|EFA09171.1| odorant receptor 13 [Tribolium castaneum]	2.00E-10	27%	6	No
Unigene21569	HassOR62	1446	409	gi|379070028|gb|AFC91725.1| putative odorant receptor OR17 [Cydia pomonella]	2.00E-88	51%	6	No
CL773.Contig1	HassOR63	1437	407	gi|512916917|ref|XP_004928758.1| PREDICTED: putative odorant receptor 85c-like [Bombyx mori]	4.00E-50	41%	7	Yes
Unigene1419	HassOR64	1371	395	gi|380011625|ref|XP_003689900.1| PREDICTED: odorant receptor 46a, isoform A-like [Apis florea]	4.00E-07	28%	6	No
Unigene13993	HassOR65	1371	422	gi|512897082|ref|XP_004924066.1| PREDICTED: uncharacterized protein LOC101736921 [Bombyx mori]	7.00E-68	70%	6	Yes
Unigene16125	HassOR66	1321	396	gi|512916917|ref|XP_004928758.1| PREDICTED: putative odorant receptor 85c-like [Bombyx mori]	1.00E-93	54%	7	Yes
Unigene2753	HassOR67	3066	398	gi|512916917|ref|XP_004928758.1| PREDICTED: putative odorant receptor 85c-like [Bombyx mori]	0	75%	7	No
CL4451.Contig1	HassOR68	1316	369	gi|512934792|ref|XP_004933118.1| PREDICTED: LOW QUALITY PROTEIN: putative odorant receptor 85b-like [Bombyx mori]	0	80%	5	No
CL1322.Contig2	HassOR69	1266	395	gi|299522734|ref|NP_001177509.1| odorant receptor 69 [Nasonia vitripennis]	3.00E-06	25%	5	No
CL2112.Contig1	HassOR70	1231	400	gi|238623687|dbj|BAH66317.1| olfactory receptor [Bombyx mori]	1.00E-128	51%	6	No
***H. armigera***								
Unigene6363	HarmOR1	1466	454	gi|148533561|gb|ABQ84982.1| putative chemosensory receptor 12 [Spodoptera littoralis]	0	85%	2	Yes
Unigene18477	HarmOR14.2	1499	440	gi|486139562|gb|AGK90006.1| olfactory receptor 14b [Helicoverpa armigera]	0	98%	4	No
CL7391.Contig1	HarmOR50	1269	400	gi|666916203|gb|AIG51896.1| odorant receptor, partial [Helicoverpa armigera]	0	98%	6	No
Unigene8351	HarmOR52	1428	419	gi|666916207|gb|AIG51898.1| odorant receptor [Helicoverpa armigera]	0	99%	6	Yes
CL3322.Contig2	HarmOR55	1515	357	gi|666916213|gb|AIG51901.1| odorant receptor, partial [Helicoverpa armigera]	8e-154	99%	3	No
Unigene26235	HarmOR56	1014	307	gi|666916215|gb|AIG51902.1| odorant receptor, partial [Helicoverpa armigera]	0	98%	1	No
Unigene15149	HarmOR57	828	264	gi|666916217|gb|AIG51903.1| odorant receptor, partial [Helicoverpa armigera]	0	99%	4	No
CL3441.Contig1	HarmOR59	1595	416	gi|666916221|gb|AIG51905.1| odorant receptor, partial [Helicoverpa armigera]	2e-173	100%	6	Yes
CL7559.Contig1	HarmOR60	1126	370	gi|666916223|gb|AIG51906.1| odorant receptor [Helicoverpa armigera]	0	98%	3	No
Unigene11361	HarmOR61	408	126	gi|669092346|gb|AII01045.1| odorant receptors [Dendrolimus houi]	7e-15	34%	2	No
Unigene16370	HarmOR62	459	142	gi|586746120|gb|EAR86837.3| transmembrane protein, putative [Tetrahymena thermophila SB210]	3.9	27%	3	No
CL1781.Contig2	HarmOR63	1481	407	gi|669092356|gb|AII01050.1| odorant receptors [Dendrolimus houi]	1e-108	41%	7	No
CL3217.Contig1	HarmOR65	1346	422	gi|512897082|ref|XP_004924066.1| PREDICTED: uncharacterized protein LOC101736921 [Bombyx mori]	2e-68	71%	6	Yes
CL252.Contig1	HarmOR66	982	296	gi|512916917|ref|XP_004928758.1| PREDICTED: putative odorant receptor 85c-like [Bombyx mori]	2e-83	56%	6	No
Unigene14307	HarmOR67	1526	398	gi|379070020|gb|AFC91721.1| putative odorant receptor OR12 [Cydia pomonella]	0	71%	7	Yes

### The candidate ionotropic receptors in *H. armigera* and *H. assulta*

**Figure 4:**
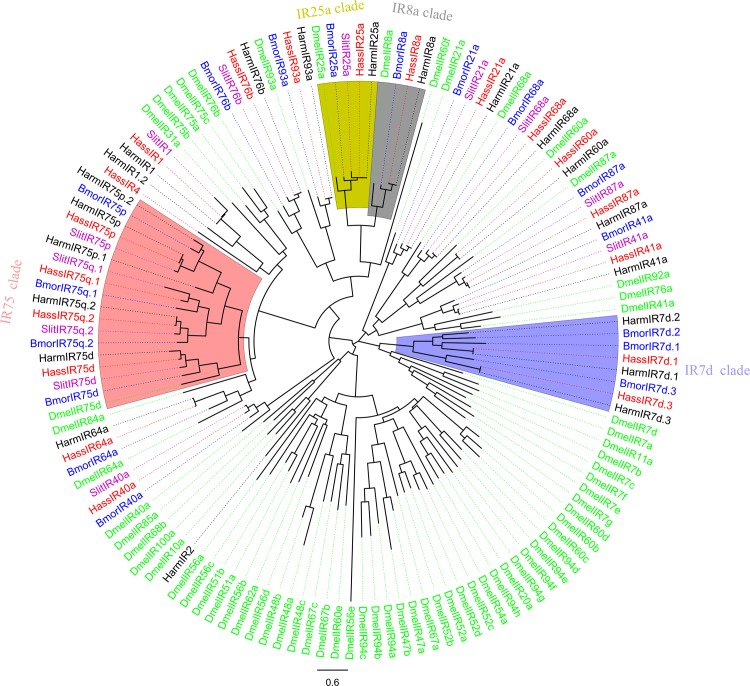
Phylogenetic tree of putative *H. armigera* and *H. assulta* IRs with IRs from other insects. This tree was constructed using FastTree based on alignment results of MAFFT. Harm: *H. armigera* (black), Hass: *H. assulta* (red), Bmor: *B. mori* (blue), Slit: *S. littoralis* (purple), Dmel: *D. melanogaster* (green).

**Figure 5:**
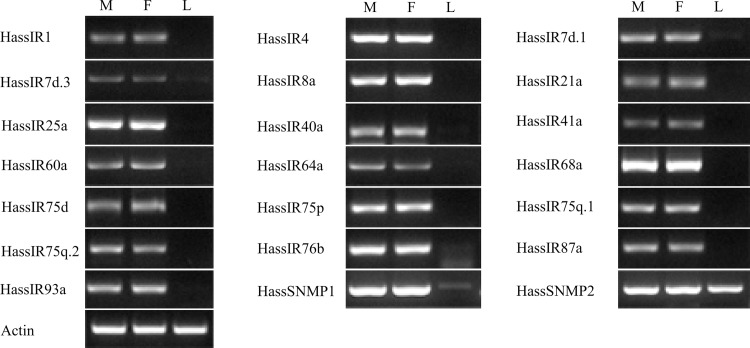
Tissue- and sex- specific expressions of *H. assulta* candidate IR and SNMP genes. M: male antennae, F: female antennae, L: legs (both sexes mixed).

A second type of olfactory receptor, IR, was first found in OSNs housed in coeloconic sensilla of *Drosophila* [47]. IRs belong to an ancient chemosensory receptor family, and most of the IRs in *Drosophila* have clear orthologs with genus of Diptera [48,49], Hymenoptera [50], and Lepidoptera [23,24,37,51]. In this study, we identified 19 IRs in *H. armigera* and 19 IRs in *H. assulta*, and named them based on homologous sequences from other insects. The 19 IRs identified in *H. assulta* and the 7 IRs not identified in previous 454 sequencing from *H. armigera* were listed here (Table 3). We did not find HarmIR7d.3 and IR75p.1 in *H. armigera* identified by Liu et al [39]. The comparison of the IR genes in *H. armigera* (from our study and Australia) and *H. assulta* were listed in S3 Material. These 19 *H. assulta* and 21 *H. armigera* IRs (including HarmIR7d.3 and IR75p.1) clustering with that of *B. mori* (17) [52], *S. littoralis* (12) [52] and *D. melanogaster* (66) [50] were used for a phylogenetic analysis (Fig. 4). The two highly conserved co-receptors IR8a and IR25a were present here as well as the two large sub-families of IR7d and IR75 clades. The phylogenetic analysis proved that there are 15 groups of orthologous IR genes in these two species, 5 *H. armigera* unique (IR1.2, IR2, IR7d.2, IR75p.1, IR75p.2) and 3 *H. assulta* unique (IR4, IR40a, IR75q.1). RT-PCR showed the IR genes were all predominantly expressed in male and female antennae (Fig. 5).

**Table 3:** Unigenes of candidate ionotropic receptors in *H. assulta* and *H. armigera*.

**Unigene reference**	**Gene name**	**Length (bp)**	**ORF (aa)**	**Blastx best hit (Reference/Name/Species)**	**E value**	**Identity**	**TMD (No)**	**Full length**
***H. assulta***								
Unigene21327	HassIR21a	2733	857	gb|ADR64678.1| putative chemosensory ionotropic receptor IR21a [Spodoptera littoralis]	0	82%	5	Yes
Unigene23288	HassIR40a	394	124	gb|ADR64680.1| putative chemosensory ionotropic receptor IR40a [Spodoptera littoralis]	5.00E-52	82%	1	No
CL1374.Contig1	HassIR41a	1926	607	gb|ADR64681.1| putative chemosensory ionotropic receptor IR41a [Spodoptera littoralis]	0	81%	3	Yes
Unigene8703	HassIR68a	1395	465	gb|ADR64682.1| putative chemosensory ionotropic receptor IR68a [Spodoptera littoralis]	0	86%	4	No
Unigene5557	HassIR75d	940	304	gb|ADR64683.1| putative chemosensory ionotropic receptor IR75d [Spodoptera littoralis]	3.00E-158	73%	0	No
CL3797.Contig2	HassIR75p	1995	619	gb|ADR64684.1| putative chemosensory ionotropic receptor IR75p [Spodoptera littoralis]	0	88%	3	Yes
CL3227.Contig1	HassIR75q.2	4384	632	gb|ADR64685.1| putative chemosensory ionotropic receptor IR75q.2 [Spodoptera littoralis]	0	82%	4	Yes
CL3227.Contig4	HassIR75q.1	2708	643	gb|ADR64686.1| putative chemosensory ionotropic receptor IR75q.1 [Spodoptera littoralis]	0	53%	3	Yes
CL3067.Contig1	HassIR76b	1947	544	gb|ADR64687.1| putative chemosensory ionotropic receptor IR76b [Spodoptera littoralis]	0	84%	3	Yes
Unigene7935	HassIR1	2165	638	gb|ADR64688.1| putative chemosensory ionotropic receptor IR1 [Spodoptera littoralis]	0	68%	3	Yes
CL4084.Contig1	HassIR87a	2270	642	gb|ADR64689.1| putative chemosensory ionotropic receptor IR87a [Spodoptera littoralis]	0	90%	3	Yes
Unigene10844	HassIR25a	3299	918	gb|AFC91757.1| putative ionotropic receptor IR25a [Cydia pomonella]	0	88%	3	Yes
CL2193.Contig4	HassIR4	2749	384	gb|AFC91763.1| putative ionotropic receptor IR4, partial [Cydia pomonella]	1.00E-46	49%	1	Yes
Unigene20831	HassIR8a	3152	895	gb|AFC91764.1| putative ionotropic receptor IR8a, partial [Cydia pomonella]	0	81%	4	Yes
Unigene7787	HassIR93a	2732	873	gb|AFC91753.1| putative ionotropic receptor IR93a, partial [Cydia pomonella]	0	75%	3	Yes
Unigene18021	HassIR7d.1	353	117	gi|666916243|gb|AIG51916.1| ionotropic receptor, partial [Helicoverpa armigera]	1e-80	99%	0	No
Unigene10598	HassIR7d.3	998	329	gi|666916247|gb|AIG51918.1| ionotropic receptor, partial [Helicoverpa armigera]	7e-91	100%	2	No
Unigene50	HassIR60a	1757	585	gi|666916249|gb|AIG51919.1| ionotropic receptor, partial [Helicoverpa armigera]	0	97%	3	No
Unigene12668	HassIR64a	1881	553	gi|666916251|gb|AIG51920.1| ionotropic receptor, partial [Helicoverpa armigera]	3e-156	97%	4	No
***H. armigera***								
Unigene36139	HarmIR2	209	69	gi|666916241|gb|AIG51915.1| ionotropic receptor, partial [Helicoverpa armigera]	3e-40	100%	1	No
Unigene30991	HarmIR7d.1	300	100	gi|666916243|gb|AIG51916.1| ionotropic receptor, partial [Helicoverpa armigera]	3e-66	100%	0	No
Unigene32554	HarmIR7d.2	353	117	gi|666916245|gb|AIG51917.1| ionotropic receptor, partial [Helicoverpa armigera]	2e-80	100%	1	No
Unigene8281	HarmIR60a	543	180	gi|666916249|gb|AIG51919.1| ionotropic receptor, partial [Helicoverpa armigera]	2e-125	99%	3	No
Unigene1966	HarmIR64a	562	187	gi|666916251|gb|AIG51920.1| ionotropic receptor, partial [Helicoverpa armigera]	8e-122	99%	2	No
Unigene4424	HarmIR68a	364	120	gi|666916253|gb|AIG51921.1| ionotropic receptor, partial [Helicoverpa armigera]	3e-81	100%	2	No
CL6604.Contig1	HarmIR93a	624	207	gi|666916257|gb|AIG51923.1| ionotropic receptor, partial [Helicoverpa armigera]	6e-132	96%	4	No

### Candidate odorant binding proteins in *H. armigera* and *H. assulta*

**Figure 6:**
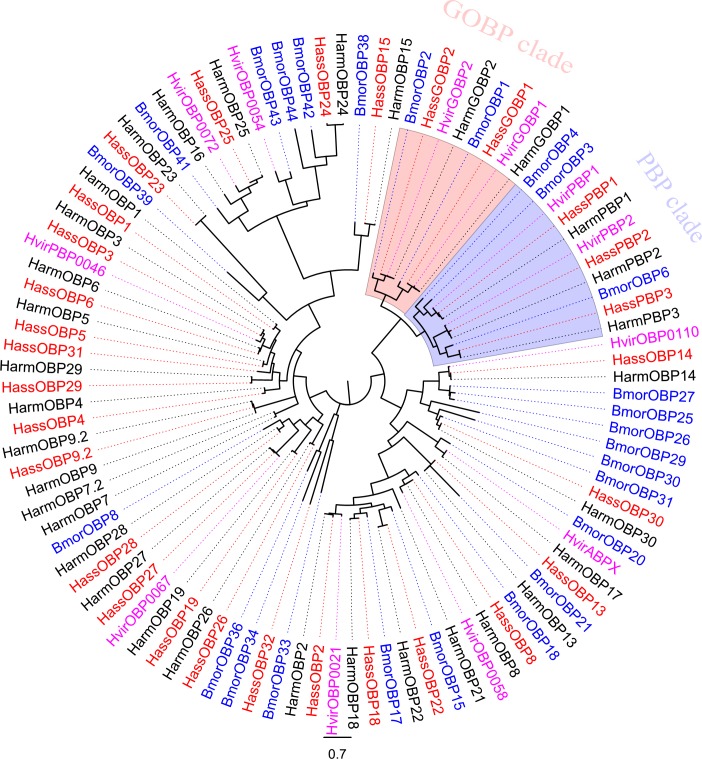
Phylogenetic tree of putative *H. armigera* and *H. assulta* OBPs with OBPs from other insects. The shown tree was constructed using FastTree based on alignment results of MAFFT. Harm: *H. armigera* (black), Hass: *H. assulta* (red), Bmor: *B. mori* (blue), Hvir: *H. virescens* (purple).

**Figure 7:**
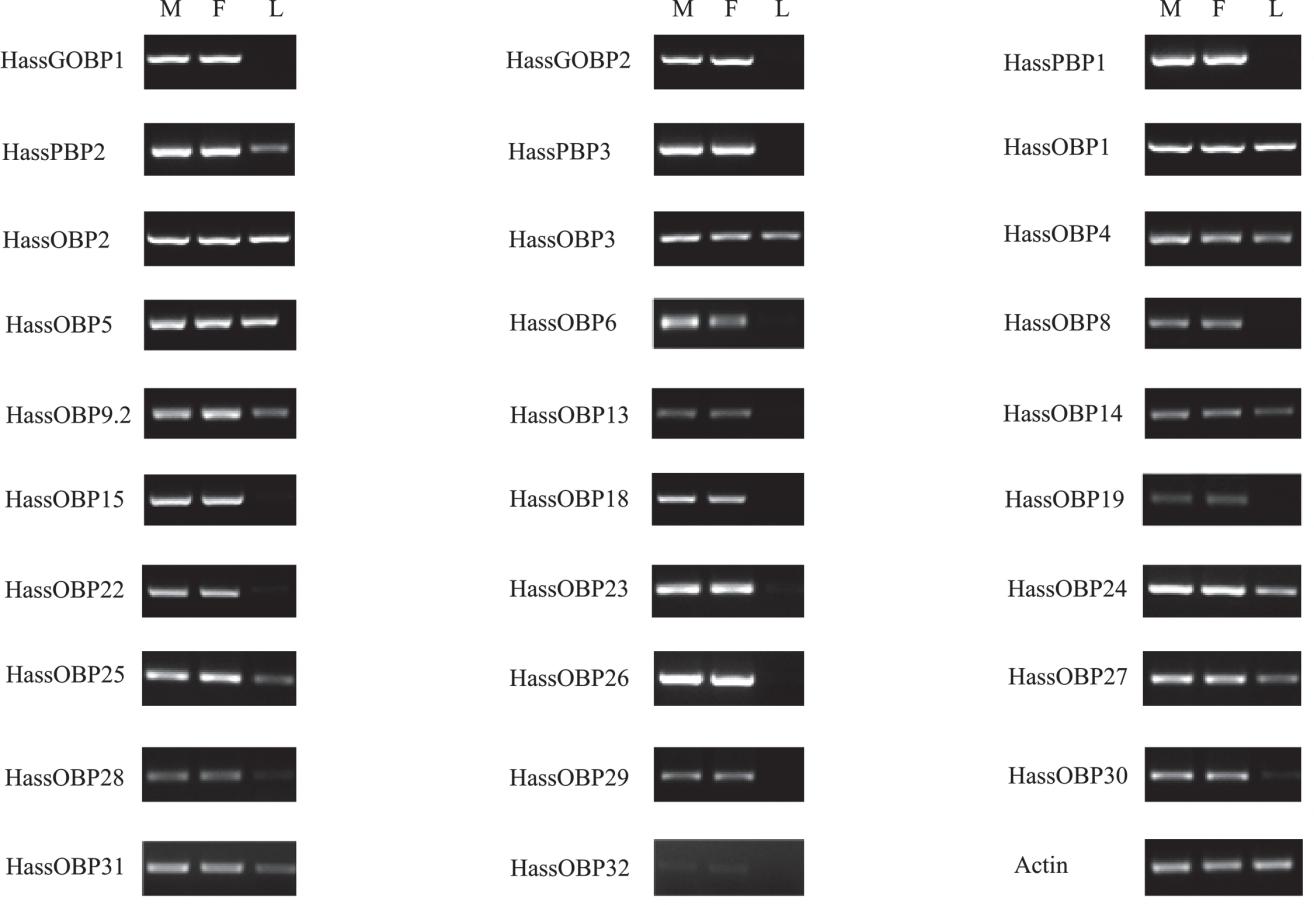
Tissue- and sex- specific expressions of *H. assulta* candidate OBP genes. M: male antennae, F: female antennae, L: legs (both sexes mixed).

OBPs are small, water-soluble, extracellular proteins that are located in the sensillum lymph, and generally thought to contribute to capture and transport of the odorants and pheromones to the receptors [16,53]. In all, we annotated 29 OBP genes from *H. assulta* and 34 OBPs genes from *H. armigera*. The 29 OBPs identified in *H. assulta* and the 8 OBPs not identified in previous 454 sequencing from *H. armigera* were listed here (Table 4). The number of OBPs identified in the present study was in a reasonable range compared to other transcriptome analyses from *Agrotis ipsilon* (33) [38], *S. littoralis* (36) [26], *S. inferens* (24) [29]. All of the 34 OBPs from *H. armigera* as well as 29 OBPs from *H. assulta* were used to construct a phylogenetic tree clustering with OBPs from *B. mori* [17] and *H. virescens* (Fig. 6) and named based on the homologous genes. The newly identified genes were named according to the length of the unigenes for HarmOBP23-30. We did not detect homologous genes (HarmOBP7, 7.2, 9, 16, 17, 20, 21) in *H. assulta*. The comparison of the OBP genes in *H. armigera* and *H. assulta* were listed in S3 Material. These differences in OBPs might be associated with differences in the recognition of volatiles emitted by the host plant, therefore functional characterization of these proteins will be a prerequisite for research in host selection. The RT-PCR results indicated that 24 *HassOBP* genes (*GOBP1, 2, PBP1, 2, 3, OBP6, 8, 9.2, 13, 14, 15, 18, 19, 22, 23, 24, 25, 26, 27, 28, 29, 30, 31* and *32*) were primarily or exclusively expressed in male and female antenna (Fig. 7). *HassOBP1, 2, 3, 4* and *5* were almost equally expressed in male and female antennae and legs. Some OBPs have been reported not being exclusive to the antenna, Sun et al reported that *PxylPBP2* and *PxylPBP3* had faint expressions in legs [54] and Sengul et al also reported that an OBP gene was expressed in male fly legs [55]. Expression of OBP56a in the oral disk of the house fly *Phormia regina* has been reported as a fatty acid solubilizer [56,57].

**Table 4:** Unigenes of candidate odorant binding proteins in *H. assulta* and *H. armigera*.

**Unigene reference**	**Gene name**	**Length (bp)**	**ORF (aa)**	**Blastx best hit (Reference/Name/Species)**	**E value**	**Identity**	**Full length**	**Signal peptide**
***H. assulta***								
**Pheromone binding protein**								
Unigene3583	HassPBP1	927	170	gb|AEB54585.1| PBP1 [Helicoverpa armigera]	1.00E-113	94%	Yes	Yes
CL1179.Contig2	HassPBP2	3054	165	gb|AEB54583.1| PBP2 [Helicoverpa armigera]	1.00E-99	92%	Yes	Yes
CL341.Contig1	HassPBP3	7544	164	gb|ABB91374.1| PBP3 [Helicoverpa assulta]	2.00E-94	100%	Yes	Yes
**General odorant binding protein**								
CL4678.Contig1	HassGOBP1	903	162	gb|AAW65076.1| general odorant binding protein 1 [Helicoverpa assulta]	6.00E-99	99%	No	Yes
CL1767.Contig2	HassGOBP2	1620	162	gb|AAQ54909.1| general odorant binding protein 2 [Helicoverpa assulta]	3.00E-109	100%	Yes	Yes
**Other odorant binding protein**								
CL126.Contig2	HassOBP1	2485	161	gb|AEX07272.1| odorant binding protein [Helicoverpa assulta]	3.00E-84	97%	Yes	Yes
CL1167.Contig1	HassOBP2	1023	143	gb|AEB54586.1| OBP2 [Helicoverpa armigera]	2.00E-76	93%	Yes	Yes
CL3124.Contig2	HassOBP3	623	147	gb|AGC92788.1| odorant binding protein 3 [Helicoverpa assulta]	4.00E-100	99%	Yes	Yes
CL1018.Contig1	HassOBP4	1008	147	gb|AEX07276.1| odorant binding protein [Helicoverpa assulta]	7.00E-85	96%	Yes	Yes
Unigene9995	HassOBP5	1624	147	gb|AEX07271.1| odorant binding protein [Helicoverpa assulta]	1.00E-92	99%	Yes	Yes
Unigene14608	HassOBP6	826	147	gb|AEX07270.1| odorant binding protein [Helicoverpa assulta]	8.00E-82	95%	Yes	Yes
Unigene16224	HassOBP8	1076	139	gb|AEB54589.1| OBP8 [Helicoverpa armigera]	5.00E-93	100%	Yes	Yes
CL3057.Contig1	HassOBP9.2	3281	148	gb|AGC92789.1| odorant binding protein 9 [Helicoverpa assulta]	7.00E-90	99%	Yes	Yes
CL3394.Contig2	HassOBP13	2390	141	gb|AGC92790.1| odorant binding protein 13 [Helicoverpa assulta]	4.00E-80	99%	Yes	Yes
CL4268.Contig1	HassOBP14	684	137	gb|AFI57167.1| odorant binding protein 18 [Helicoverpa armigera]	2.00E-87	99%	Yes	Yes
Unigene19151	HassOBP15	816	168	gb|ADY17882.1| odorant binding protein [Spodoptera exigua]	2.00E-74	73%	Yes	Yes
CL1135.Contig1	HassOBP18	2355	146	gb|AFG72998.1| odorant binding protein 1 [Cnaphalocrocis medinalis]	3.00E-69	79%	Yes	Yes
CL2004.Contig3	HassOBP19	1810	130	gb|AGP03460.1| SexiOBP14 [Spodoptera exigua]	2.00E-49	62%	Yes	No
CL810.Contig2	HassOBP22	7234	146	gb|AGP03458.1| SexiOBP12 [Spodoptera exigua]	6.00E-69	76%	Yes	Yes
CL2229.Contig1	HassOBP23	1763	244	gb|AGH70107.1| odorant binding protein 11 [Spodoptera exigua]	2.00E-68	81%	Yes	Yes
CL2035.Contig1	HassOBP24	700	204	gb|AGC92793.1| odorant binding protein 19 [Helicoverpa assulta]	8.00E-123	99%	Yes	Yes
CL1769.Contig1	HassOBP25	658	186	gb|AEX07273.1| odorant binding protein [Helicoverpa assulta]	2.00E-128	97%	Yes	Yes
Unigene15089	HassOBP26	1415	154	gb|AGP03457.1| SexiOBP11 [Spodoptera exigua]	6.00E-80	76%	Yes	Yes
Unigene2050	HassOBP27	584	147	gb|AEX07279.1| odorant binding protein [Helicoverpa armigera]	1.00E-85	97%	Yes	Yes
Unigene4231	HassOBP28	664	147	gb|AFM77984.1| oderant binding protein 6 [Spodoptera exigua]	1.00E-56	59%	Yes	Yes
CL965.Contig1	HassOBP29	2226	142	gb|AGP03459.1| SexiOBP13 [Spodoptera exigua]	1.00E-38	53%	Yes	Yes
CL4245.Contig1	HassOBP30	815	135	gb|AFI57166.1| odorant-binding protein 17 [Helicoverpa armigera]	3.00E-90	99%	Yes	Yes
Unigene18647	HassOBP31	410	125	gb|AEB54581.1| OBP5 [Helicoverpa armigera]	3.00E-44	61%	No	Yes
Unigene20693	HassOBP32	413	124	gb|AHA33381.1| odorant-binding protein 3 [Batocera horsfieldi]	3.00E-05	32%	No	Yes
***H. armigera***								
Unigene6066	HarmOBP23	1745	244	gb|AGH70107.1| odorant binding protein 11 [Spodoptera exigua]	1e-91	80%	Yes	Yes
Unigene19842	HarmOBP24	741	201	gb|AGC92793.1| odorant binding protein 19 [Helicoverpa assulta]	1e-127	95%	Yes	Yes
CL4113.Contig1	HarmOBP25	605	195	gb|AEX07273.1| odorant binding protein [Helicoverpa assulta]	3e-131	98%	Yes	Yes
Unigene6168	HarmOBP26	579	154	gb|AGP03457.1| SexiOBP11 [Spodoptera exigua]	2e-84	75%	Yes	Yes
Unigene30181	HarmOBP27	305	99	gb|AEX07279.1| odorant binding protein [Helicoverpa armigera]	2e-60	95%	No	Yes
Unigene13719	HarmOBP28	620	147	gb|AFM77984.1| oderant binding protein 6 [Spodoptera exigua]	1e-57	59%	Yes	Yes
CL1886.Contig1	HarmOBP29	569	142	gb|AGP03459.1| SexiOBP13 [Spodoptera exigua]	3e-46	57%	Yes	Yes
Unigene21059	HarmOBP30	466	131	gb|AFI57166.1| odorant-binding protein 17 [Helicoverpa armigera]	8e-90	99%	Yes	Yes

### Candidate chemosensory proteins in *H. armigera* and *H. assulta*

**Figure 8:**
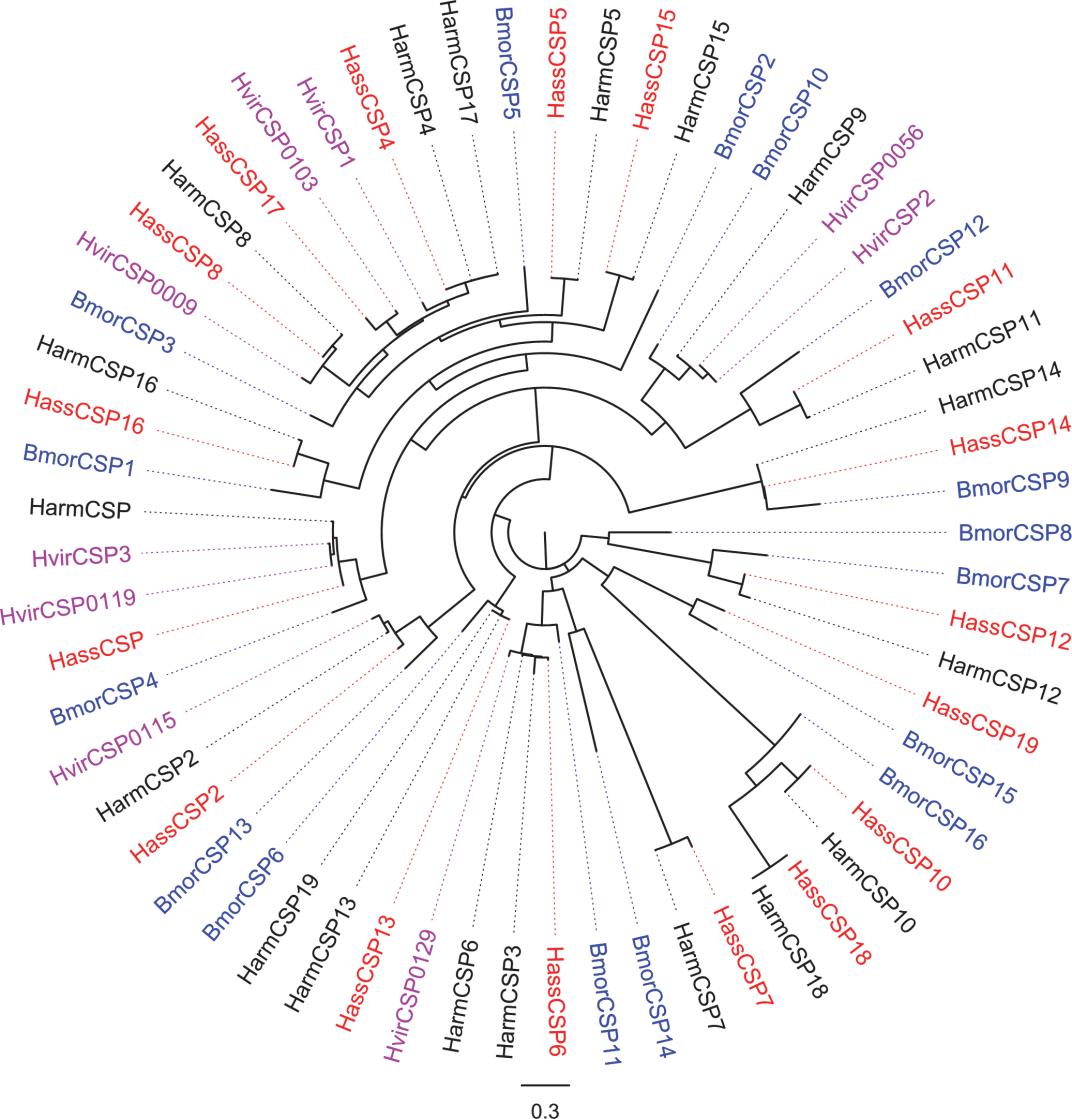
Phylogenetic tree of putative *H. armigera* and *H. assulta* CSPs with CSPs from other insects. The shown tree was constructed using FastTree based on alignment results of MAFFT. Harm: *H. armigera* (black), Hass: *H. assulta* (red), Harm: *H. armigera* (green), Bmor: *B. mori* (blue), Hvir: *H. virescens* (purple).

**Figure 9:**
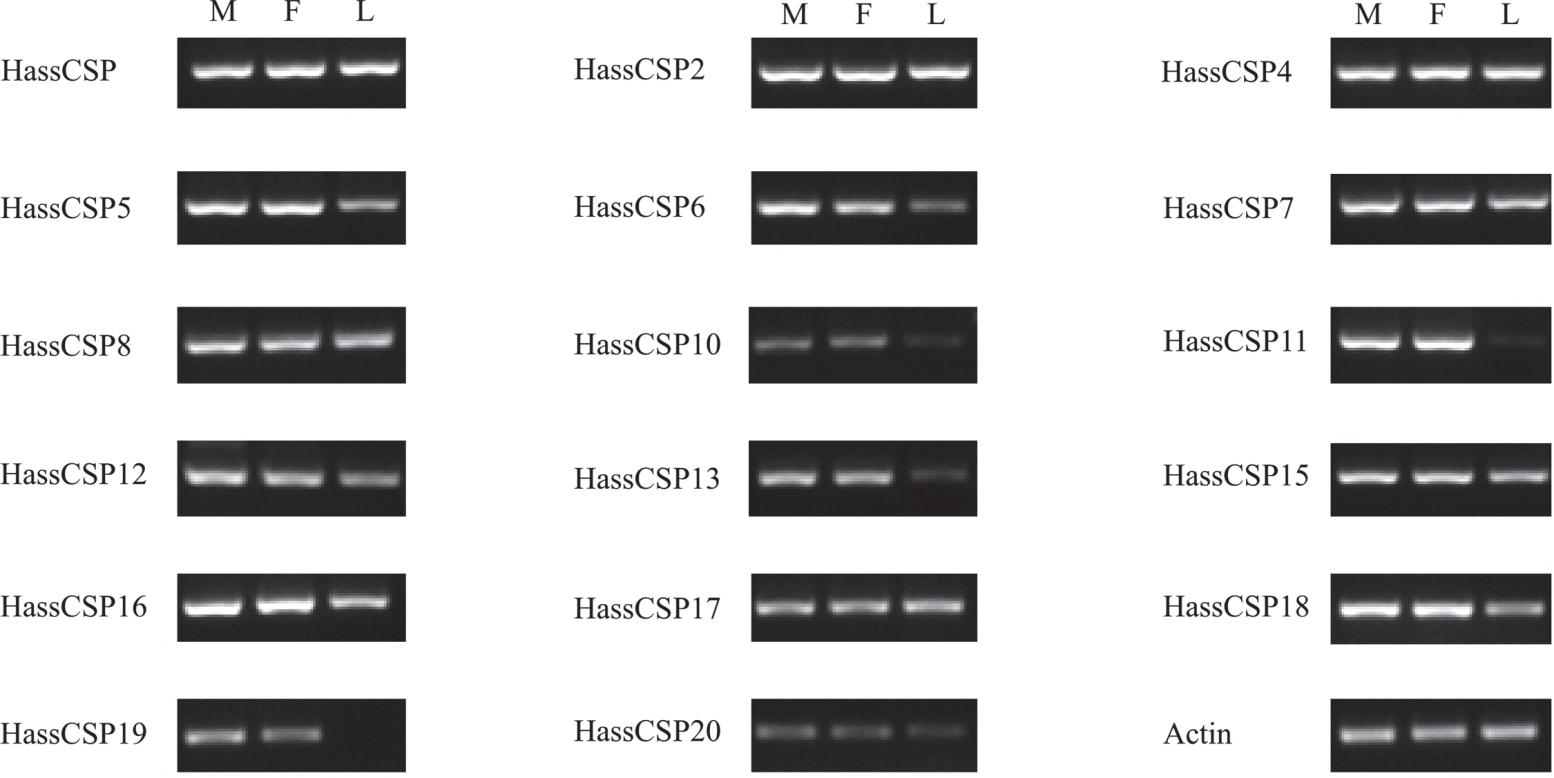
Tissue- and sex- specific expressions of *H. assulta* candidate CSP genes. M: male antennae, F: female antennae, L: legs (both sexes mixed).

CSPs are another class of small soluble proteins in insects. Several CSPs are present at high concentrations in the lymph of chemosensilla and exhibit binding activity towards odorants and pheromones [58]. In this study, we identified 17 CSP genes in *H. assulta* and 18 CSP genes in *H. armigera*. The 17 CSPs identified in *H. assulta* and the 6 CSPs not identified in previous 454 sequencing from *H. armigera* were listed here (Table 5). The comparison of the CSP genes in *H. armigera* and *H. assulta* were listed in S3 Material. All of the 17 CSPs from *H. assulta* and 18 CSPs from *H. armigera* were used to construct a phylogenetic tree clustering with CSPs from *B. mori* [59] and *H. virescens* [60] (Fig. 8) and named based on the homologous genes, while the newly identified genes HarmCSP14-19 were named according to the length of the unigenes. We did not detect the homologous gene of HarmCSP9 in *H. assulta*. Comparing with the number of CSPs identified from other Lepidoptera: *B. mori* (18) [61], *M. sexta* (21) [23], *S. littoralis* (21) [26], *S. inferens* (24) [37], we may have missed some CSPs in this transcriptome. The RT-PCR results showed that *CSP6, 10, 11, 13, 19* were antennal enriched, while other *CSPs* were almost equally expressed in the three test tissues (Fig. 9). The antennal enriched CSPs may be involved in the chemoreception [62]. There are some reports about the expression of CSPs in tissues other than antenna [37,63–65]. The CSPs expressed in legs may participate in other physiological processes beyond chemoreception [66].

**Table 5:** Unigenes of candidate chemosensory proteins in *H. assulta* and *H. armigera*.

**Unigene reference**	**Gene name**	**Length (bp)**	**ORF (aa)**	**Blastx best hit (Reference/Name/Species)**	**E value**	**Identity**	**Full length**	**Signal peptide**
***H. assulta***								
CL2680.Contig1	HassCSP	774	127	gb|ABB91378.1| chemosensory protein [Helicoverpa assulta]	6.00E-73	98%	Yes	Yes
CL4029.Contig1	HassCSP2	407	120	gb|AEX07265.1| CSP2 [Helicoverpa armigera]	3.00E-75	91%	Yes	Yes
Unigene16387	HassCSP4	1640	96	gb|AEX07269.1| CSP4 [Helicoverpa armigera]	4.00E-46	100%	No	No
CL2023.Contig1	HassCSP5	757	127	gb|AEB54579.1| CSP5 [Helicoverpa armigera]	9.00E-85	99%	Yes	Yes
CL3762.Contig1	HassCSP6	1227	123	gb|AEX07267.1| CSP6 [Helicoverpa armigera]	8.00E-76	97%	Yes	Yes
CL1078.Contig1	HassCSP7	1305	111	gb|AEX07268.1| CSP7 [Helicoverpa armigera]	1.00E-63	93%	Yes	Yes
CL488.Contig1	HassCSP8	543	128	gb|AFR92095.1| chemosensory protein 11 [Helicoverpa armigera]	3.00E-76	91%	Yes	Yes
Unigene16790	HassCSP10	692	107	gb|AGR39575.1| chemosensory protein 5 [Agrotis ipsilon]	1.00E-54	92%	Yes	Yes
CL126.Contig12	HassCSP11	3000	150	gb|AGY49270.1| putative chemosensory protein [Sesamia inferens]	2.00E-77	84%	Yes	Yes
Unigene8153	HassCSP12	361	102	gb|AFR92092.1| chemosensory protein 8 [Helicoverpa armigera]	1.00E-63	93%	No	Yes
Unigene20980	HassCSP13	683	122	gb|AFR92094.1| chemosensory protein 10 [Helicoverpa armigera]	4.00E-82	100%	Yes	Yes
CL3149.Contig1	HassCSP14	1914	292	gb|AHC05676.1| chemosensory protein [Chilo suppressalis]	4.00E-59	77%	Yes	Yes
CL2815.Contig2	HassCSP15	477	124	gb|AGH20053.1| chemosensory protein 15, partial [Helicoverpa armigera]	1.00E-72	98%	Yes	Yes
Unigene18506	HassCSP16	520	123	gb|AGR39578.1| chemosensory protein 8 [Agrotis ipsilon]	5.00E-57	77%	Yes	Yes
Unigene215	HassCSP17	434	110	gb|AGH20056.1| chemosensory protein 18, partial [Helicoverpa assulta]	3.00E-60	99%	No	No
Unigene18603	HassCSP18	482	107	gb|AGY49260.1| putative chemosensory protein, partial [Sesamia inferens]	5.00E-44	99%	Yes	Yes
Unigene26196	HassCSP19	363	101	ref|NP_001091781.1| chemosensory protein 15 [Bombyx mori]	1.00E-39	68%	No	No
***H. armigera***								
CL4900.Contig1	HarmCSP14	2257	292	gb|KF487617.1| chemosensory proteins 5 [Dendrolimus houi]	4.00E-80	79%	Yes	Yes
Unigene9880	HarmCSP15	562	124	gi|461726399|gb|AGH20053.1| chemosensory protein 15, partial [Helicoverpa armigera]	5e-72	99%	Yes	Yes
Unigene24782	HarmCSP16	517	123	gb|AGR39578.1| chemosensory protein 8 [Agrotis ipsilon]	2.00E-56	76%	Yes	Yes
CL1141.Contig1	HarmCSP17	524	128	gb|AEX07269.1| chemosensory protein 4 [Helicoverpa armigera ]	1.00E-75	99%	Yes	Yes
Unigene26004	HarmCSP18	526	107	gb|AGY49260.1| putative chemosensory protein, partial [Sesamia inferens]	1.00E-43	99%	Yes	Yes
CL5007.Contig1	HarmCSP19	1082	122	gb|AFR92094.1| chemosensory protein 10 [Helicoverpa armigera ]	5.00E-80	100%	Yes	Yes

### Candidate sensory neuron membrane proteins in the *H. assulta*

SNMPs are insect membrane proteins that are associated with pheromone-sensitive neurons in Lepidoptera and Diptera [67–70]. The insect SNMP family consists of two subfamilies, SNMP1 and SNMP2, which were first identified from *A. polyphemus* [67] and *Manduca sexta* [71], respectively. Since then, much progress has been achieved in the identification of SNMP1 and SNMP2 in different insect orders [69,72–80]. The expression of SNMP1 in pheromone-specific olfactory neurons suggests it may be involved in pheromone detection [44,67,72], while SNMP2 was found to express in the supporting cells [70,77]. In this study, we identified two SNMP genes, SNMP1 and SNMP2, from *H. assulta* antennal transcriptomes (Table 6), for the identification of two SNMP in our previous study, we did not list the HarmSNMP here. Both HassSNMP1 and SNMP2 have the complete ORFs and the two transmembrane domains. RT-PCR results showed *SNMP1* was primarily expressed in antennae and *SNMP2* was aboundant expressed in antennae as well as in legs (Fig. 5).

**Table 6:** Unigenes of candidate sensory neuron membrane protein in *H. assulta*.

**Unigene reference**	**Gene name**	**Length (bp)**	**ORF (aa)**	**Blastx best hit (Reference/Name/Species)**	**E value**	**Identity**	**Full length**	**TMD (No)**
Unigene4312	HassSNMP1	2876	523	gi|510381297|gb|AGN48098.1| sensory neuron membrane protein 1 [Spodoptera litura]	0	88%	Yes	2
Unigene1683	HassSNMP2	1742	520	gi|510812726|gb|AGN52677.1| sensory neuron membrane protein 2 [Spodoptera exigua]	0	86%	Yes	2

## Conclusion

We carried out comprehensive analysis of the antennal transcriptomes of *H. armigera* and *H. assulta* using Illumina HiSeq 2000 platform. We successfully annotated 133 putative chemosensory genes: 60 ORs, 19 IRs, 34 OBPs, 18 CSPs, and 2 SNMPs in *H. armigera* and 131 putative chemosensory genes: 64 ORs, 19 IRs, 29 OBPs, 17 CSPs, and 2 SNMPs in *H. assulta*. Later we completely compared the differences of the chemosensory genes between these two species. RT-PCR confirmed the distribution profiles of these chemosensory genes in different tissues and found a predominance of expression in the male and female antennae. This transcriptomic analyses greatly expands the information in *H. armigera* compared to the 454 sequencing and will surely benefit the exploration of chemoreception mechanisms in *H. armigera* and *H. assulta*.

## Materials and Methods

### Insect rearing and tissue collection

The *H. assulta* larvae were collected from the tobacco fields with the permission of the Experiment Station of Henan University of Science and Technology in Xuchang, Henan Province, China. The insects were fed with an artificial diet at a temperature of 27 ± 1°C with a photoperiod of 16 h: 8 h, L: D. Pupae were sexed and males and females were put into separate cages for eclosion. *H. armigera* used in all experiments were obtained from a colony maintained at the Institute of Plant Protection, Chinese Academy of Agricultural Sciences, Beijing, China. Adult moths were given a 10% honey solution after emergence. Antennae were excised from 3-day-old male and female moths and immediately frozen and stored in liquid nitrogen until use.

### cDNA Library Construction and Illumina Sequencing

Total RNA was extracted using TRIzol reagent (Invitrogen, Carlsbad, CA, USA). Total RNA was dissolved in RNase-free water and RNA integrity was verified by gel electrophoresis. RNA quantity was determined on a Nanodrop ND-2000 spectrophotometer (NanoDrop products, Wilmington, DE, USA). cDNA library construction and Illumina sequencing of the samples were performed at Beijing Genomics Institute (BGI, Shenzhen, China). To disrupt mRNA into short fragments, fragmentation buffer and divalent cations were used at 94°C for 5 min. Using these short fragments as templates, random hexamer-primers were used to synthesize first-strand cDNA. Second-strand cDNA was generated using buffer, dNTPs, RNase H, and DNA polymerase I. After end-repair and ligation of adaptors, the products were amplified by PCR and purified with QIAquick PCR purification kit (Qiagen, Hilden, Germany) and resolved with elution buffer (EB) for end reparation and poly (A) addition. Then, the short fragments connected to sequencing adapters and detected by agarose gel electrophoresis were selected as templates for PCR amplification and sequencing using Illumina HiSeq 2000 (Illumina, San Diego, CA, USA).

### Assembly and function annotation

Transcriptome de novo assembly was carried out with the short read assembly program Trinity (version 20120608) [81]. Then the Trinity outputs were clustered by TGICL [82]. The consensus cluster sequences and singletons make up the unigenes dataset. The annotation of unigenes were performed by NCBI blastx against a pooled database of non-redundant (nr) and SwissProt protein sequences with e-value < 1e-5. The blast results were then imported into Blast2GO [83] pipeline for GO Annotation.

### Identification of chemosensory genes

The tblastn program was performed, with available sequences of OBP, CSP, OR, IR and SNMP proteins from Lepidoptera species as “query” to identify candidate unigenes encoding putative OBPs, CSPs, ORs, IRs and SNMPs in *H. armigera* and *H. assulta*. All candidate OBPs, CSPs, ORs, IRs and SNMPs were manually checked by the blastx program at the National Center for Biotechnology Information (NCBI).

### Sequence and phylogenetic analysis

The open reading frames (ORFs) of the putative chemosensory genes were predicted by using ORF finder (http://www.ncbi.nlm.nih.gov/gorf/gorf.html). Putative N-terminal signal peptides of OBPs and CSPs were predicted by Signal IP 4.0 (http://www.cbs.dtu.dk/services/SignalP/) [84]. The TMDs (Transmembrane Domain) of ORs and IRs were predicted using TMHMM Server Version 2.0 (http://www.cbs.dtu.dk/services/TMHMM). Alignments of amino acid sequences were performed by MAFFT (https://www.ebi.ac.uk/Tools/msa/mafft/). The phylogenetic trees were constructed by FastTree for phylogenetic analyses of ORs, IRs, OBPs, and CSPs, based on the amino acid sequences of the putative chemosensory genes and the sequences of other insects. The OR data set contained OR sequences identified in Lepidoptera (64 from *H. assulta*, 66 from *H. armigera* (including HarmOR4, 49, 51, 53, 54, 58 identified by Liu et al), 21 from *H. virescens* and 62 from *B. mori*) [24,39,40,43,44]. The IR data set contained 19 IRs from *H. assulta*, 21 IRs from *H. armigera* (including HarmIR7d.3 and IR75p.1 identified by Liu et al), 12 IRs from *S. littoralis*, 17 IRs from *B. mori* and 66 IRs from *D. melanogaster* [24,39,50,52]. The OBP data set contained OBP sequences 34 from *H. armigera*, 29 from *H. assulta*, 35 from *B. mori* and 17 from *H. virescens* [17,24,39]. The CSP data set contained the 18 sequences from *H. armigera*, 17 sequences from *H. assulta*, 16 sequences from *B. mori* [59] and 9 from *H. virescens* [60].

### Expression analysis by semi-quantitative reverse transcription PCR

Semi-quantitative reverse transcription PCR was performed to verify the expression of candidate chemosensory genes. Male and female antennae, legs (both sexes mixed) were collected from 3-day olds adult *H. assulta* after eclosion and total RNA was extracted using TRIzol reagent (Invitrogen, Carlsbad, CA, USA) and digested with DNase I (Fermentas, Vilnius, Lithuania). The cDNA was synthesized from total RNA using RevertAid First Strand cDNA Synthesis Kit (Thermo Scientific, Waltham, MA, USA). Gene specific primers were designed using Primer 3 (http://www.ncbi.nlm.nih.gov/tools/primer-blast/) (S2 Material) and synthesized by Sangon Biotech Co., Ltd (Shanghai, China). Taq MasterMix (CWBIO, Beijing, China) was used for PCR reactions under general 3-step amplification of 94°C for 30s, 55–60°C for 30s, 72°C for 30s.

## Supporting Information

S1 Material Species distribution of the blastx results. Unigenes were searched against the NR protein database using blastx with a cut off e-value < 10^−5^. Species with proportions of more than 1% are shown.
(TIF)

S2 Material Primers for RT-PCR expression analyses of *H. assulta* ORs, IRs, OBPs, CSPs, SNMPs. (XLS)

S3 Material The complete comparison of the olfactory genes (ORs, IRs, OBPs, CSPs, SNMPs) in *H. armigera* (from our study and Australia) and *H. assulta*. (XLSX)
