# Supplementary figures and images for: Antennal Transcriptome Analysis and Comparison of Chemosensory Gene Families in Two Closely Related Noctuidae Moths, Helicoverpa armigera and H. assulta
Source: PLoS One. 2015 Feb 6;10(2):e0117054. doi: 10.1371/journal.pone.0117054 (PMC4319919; doi:10.1371/journal.pone.0117054)

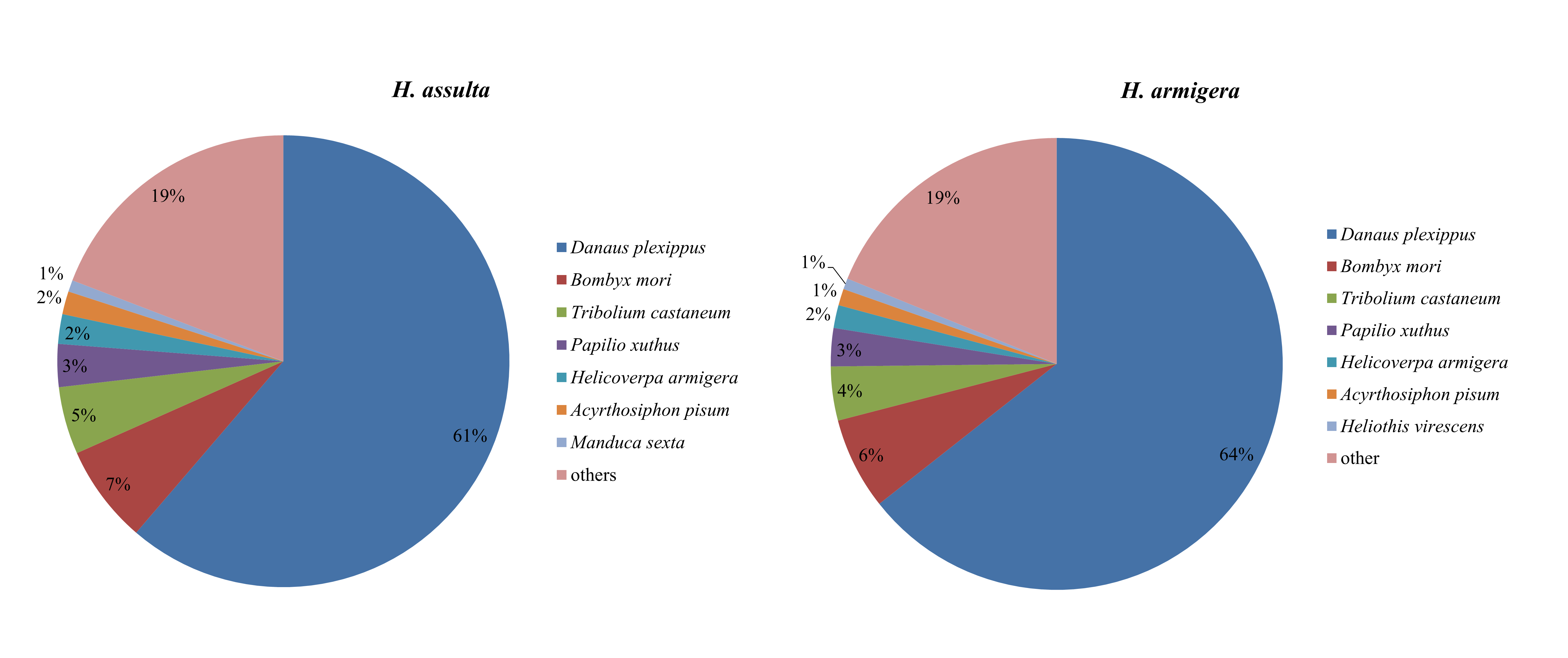

Supplement: S1 Material — Species with proportions of more than 1% are shown. (TIF) [file pone.0117054.s001.tif]
